# Receptor chimeras demonstrate that the C-terminal domain of the human cytomegalovirus US27 gene product is necessary and sufficient for intracellular receptor localization

**DOI:** 10.1186/1743-422X-9-42

**Published:** 2012-02-16

**Authors:** Lance K Stapleton, Kathleen L Arnolds, Angela P Lares, Tori M Devito, Juliet V Spencer

**Affiliations:** 1 Department of Biology, University of San Francisco, 2130 Fulton Street, Harney Science Center Room 342, San Francisco, CA 94117, USA

**Keywords:** HCMV, Cytomegalovirus, Chemokines, Chemokine receptors, GPCR, US27

## Abstract

**Background:**

Human cytomegalovirus (HCMV) is ubiquitous in the population but generally causes only mild or asymptomatic infection except in immune suppressed individuals. HCMV employs numerous strategies for manipulating infected cells, including mimicry of G-protein coupled receptors (GPCRs). The HCMV US27 gene product is a putative GPCR, yet no ligand or signaling has been identified for this receptor. In the present study, immunofluorescence microscopy was used to examine the cellular distribution of wild type US27, as well as US27 deletion mutants and chimeric receptors.

**Results:**

In transiently transfected cells, wild type US27 was found primarily in intracellular compartments, in striking contrast to the cell surface distribution seen for the human cellular chemokine receptor CXCR3. When the N-terminal extracellular domains of the two receptors were swapped, no change in protein localization was observed. However, swapping of the C-terminal intracellular domains resulted in a significant change in receptor distribution. A chimera that contained US27 fused to the C-terminal intracellular tail of CXCR3 exhibited surface distribution similar to that of wild-type CXCR3. When the C-terminal domain of US27 was fused to CXCR3, this chimeric receptor (CXCR3/US27-CT) was found in the same intracellular pattern as wild-type US27. In addition, a US27 mutant lacking the C-terminus (US27ΔCT) failed to accumulate inside the cell and exhibited cell surface distribution. Co-localization with organelle-specific markers revealed that wild-type US27 was found predominantly in the Golgi apparatus and in endosomal compartments, whereas the US27/CXCR3-CT chimera, US27ΔCT and US27Δ348 mutants were not localized to endosomal compartments.

**Conclusions:**

The results indicate that the C-terminal domain of the HCMV US27 protein, which contains a di-leucine endocytic sorting motif, is both necessary and sufficient for intracellular localization, which may also help explain why no cellular ligands have yet been identified for this viral receptor.

## Background

Human cytomegalovirus (HCMV) is a herpesvirus that is ubiquitous in the population with a seroprevalence of 50-95% [1]. Primary infection is usually asymptomatic, but more serious disease can occur in the immune-compromised patient. HCMV pneumonitis greatly impacts the morbidity and mortality of transplant recipients, and increasing numbers of HIV patients have been diagnosed with severe HCMV retinitis [2]. HCMV can also be transmitted from mother to child during pregnancy and remains the most common viral cause of congenital defects, including deafness and mental retardation [3].

In the healthy host, HCMV maintains a successful co-existence facilitated by numerous mechanisms that the virus has acquired for modulating the host immune response [4]. The virally encoded US2, US3, US6 and US11 gene products all interfere with antigen processing and presentation, resulting in reduced major histocompatibility complex (MHC) class I presentation [5], and thus decreased recognition by cytotoxic T lymphocytes. In addition, the UL18 gene product is a homolog of the MHC class I protein that is postulated to act as a decoy on the cell surface to assist in the evasion of natural killer cells [6]. A homolog of interleukin-10 encoded by UL111A was found to inhibit inflammatory cytokine secretion [7,8], and an α-chemokine encoded by UL146 has been shown to stimulate neutrophil migration [9]. In addition, the HCMV genome encodes four putative GPCRs containing seven transmembrane domains and similarity to human chemokine receptors: US27, US28, UL33, and UL78 [10]. Chemokines are a class of cytokines important in their regulation of motility and activation of immune cells, and the existence of viral homologs of chemokine receptors indicates additional mechanisms for manipulation of the host immune response.

The US28 gene product is perhaps the most extensively characterized viral GPCR; it is expressed early in infection and has been shown to bind to a broad range of human chemokines, including fractalkine, RANTES, and MIP-1α [11-14]. The US28 receptor couples to a variety of G proteins, including Gα_q_, Gα_i/o_, Gα_12_, and Gα_16 _[12,15,16]. In in vitro assay systems with various cell types expressing US28, chemokine binding has been shown to induce an assortment of intracellular responses, including calcium mobilization, MAP kinase activation, cell migration, and activation of transcription factors, such as NFAT, CREB, and NF-κB [11-13,15,17]. Ligand-independent signaling has also been observed for the US28 receptor; constitutive phospholipase C activation and inositol phosphate (IP_3_) production occur in transfected Cos-7 and Hela cells expressing US28 [12,18]. Expression of US28 transcripts occurs throughout the infection cycle, at immediate early, early, and late time points [14,19], as well as during latency in THP-1 monocytes [20]. Although no homologs of this receptor exist in the genomes of rodent CMVs, five tandem homologs of US28 have been identified in the genome of Rhesus CMV [21]. The US28 protein is a versatile viral receptor with the ability to alter signaling networks during infection in many different ways.

The UL33 gene of HCMV is highly conserved among herpesviruses, with homologs in rat CMV (RCMV R33) and murine CMV (MCMV M33), as well as the U12 genes of HHV-6 and HHV-7. UL33, R33, and M33 have all been found to display constitutive signaling activity, as evidenced by modulation of CRE (cAMP response element)-mediated transcription [22,23]. To date, no ligands have been identified for these receptors, and the UL33 homologs from all CMVs remain orphans. Although UL33, R33, and M33 are all dispensable for virus replication in vitro [24-26], the biological significance of UL33 family members has been demonstrated in in vivo studies. A deletion mutant of MCMV lacking functional M33 demonstrated impaired replication in the salivary glands of infected mice [25,27], and M33 is widely considered to be a functional homolog of HCMV US28. Likewise, when immune-compromised rats were infected with a recombinant RCMV strain lacking R33, no virus replication was observed in the salivary glands and animals infected with the mutant strain had lower mortality rates than those infected with wild-type virus [26], suggesting that this protein plays a role in pathogenesis in vivo.

The initial analysis of the HCMV genome predicted the presence of only three GPCRs, US27, US28, and UL33 [28]. Subsequent analysis defined a fourth putative GPCR, UL78, based on similarity to an HHV-6 gene [29]. Microarray analysis of HCMV-infected fibroblasts indicates that UL78 is expressed at the early stage of infection [30]. Homologs of UL78 exist in the genomes of the rodent CMVs. MCMV M78 exists as a dimer that is rapidly endocytosed [31]; the gene is transcribed at early times in infected cells and was also found to be present in the virus envelope [32]. In contrast, RCMV R78 is expressed both early and late in infection [33]. While UL78 is not required for HCMV replication in vitro, as demonstrated by the unimpaired growth of null mutants in culture [34], mutant RCMV strains lacking R78 have a syncytium-inducing phenotype in rat endothelial fibroblasts and cause reduced mortality in infected rats [33]. R78, like R33, appears to impact in vivo viral pathogenesis.

US27 remains the least studied of the HCMV GPCRs, and no homologs have been described for CMV strains infecting other species. US27 is directly adjacent to US28 in the HCMV genome and the two proteins share 31% sequence identity, leading to speculation that a gene duplication event may have occurred. Although it has not yet been shown to be a functional receptor, the US27 gene product contains several features that are characteristic of human chemokine receptors, such as the seven-transmembrane α-helical domains. While overall sequence homology among most GPCRs is low, certain key residues tend to be highly conserved. For instance, the DRY motif (aspartic acid-arginine-tyrosine) is critical for receptor activation and stabilization [35], and that motif is present in the second intracellular loop of US27. The second and third extracellular domains of US27 contain two conserved cysteine residues (C104 and C176, respectively) that have been shown to be essential for structure and ligand binding for many GPCRs [28,36,37]. In addition, the extracellular domain of US27 has been shown to be heavily glycosylated [38], a common characteristic among receptors that have small peptide or chemokine ligands [39]. Although neither ligands nor any ligand-independent constitutive signaling [22] have been identified for this receptor so far, these observations suggest that HCMV US27 may be involved in the modification of host chemokine signaling. The US27 gene product exhibits greatest amino acid sequence identity with US28, followed closely by human chemokine receptors CCR1 and CCR4 (29% sequence identity for each).

US27 is expressed late in infection [40], and the 2.9 kb transcript was found to be spliced within the 5' untranslated region, a possible indication that post-transcriptional regulation of US27 gene expression occurs [41]. Both UL33 and US27 have been shown to be present in the virion particle and in endocytic membrane compartments of infected cells, suggesting that they may play a role during virus infection [24,38,42]. Viral mutants that lack the US27 gene are replication competent [14]; however, a slight growth defect was observed at low multiplicities of infection compared to wild type virus [43]. While the US27 deficient virus could spread directly from cell to cell, the mutant failed to spread by an extracellular route. Interestingly, no replication deficiency was noted in a virus lacking both US27 and US28 [14].

Although US27 is not required for lytic virus infection in vitro, this does not preclude an important role for this receptor in subverting the host immune response in vivo. In this study, the US27 protein was expressed in mammalian cells. Due to the predominantly intracellular localization noted here and by others [42], a series of deletion mutants and chimeric receptors were created in order to identify specific protein domains required for the subcellular distribution. We found that the C-terminal intracellular domain contained sequences that were both necessary and sufficient for directing US27 to the endosomes.

## Results

**Figure 1:**
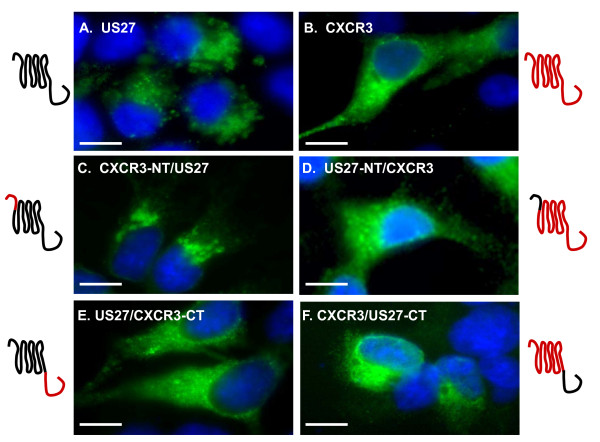
**Immunofluorescence images of cells expressing US27 and US27 chimeras**. HEK293 cells were seeded onto glass coverslips, transfected with the indicated pcDNA constructs, and then fixed and stained with anti-myc antibodies. After treatment with FITC-conjugated anti-mouse secondary antibodies, the coverslips were mounted with Prolong Gold containing DAPI. Line drawings beside each photo depict each 7TM receptor and the chimeras that were made using domains of US27 (black) and CXCR3 (red). Scale bars = 10 μm.

**Figure 2:**
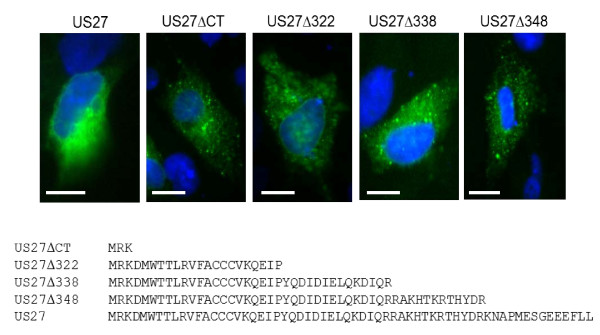
**Expression of US27 deletion mutants in transfected cells**. HEK293 cells were seeded onto glass coverslips, transfected with the indicated p3XFLAG constructs, and then fixed and stained with permeabilization with anti-FLAG antibodies as described. Scale bars = 10 μm. The amino acid sequence of the C-terminal intracellular domain of US27 and each truncation mutant is shown.

**Figure 3:**
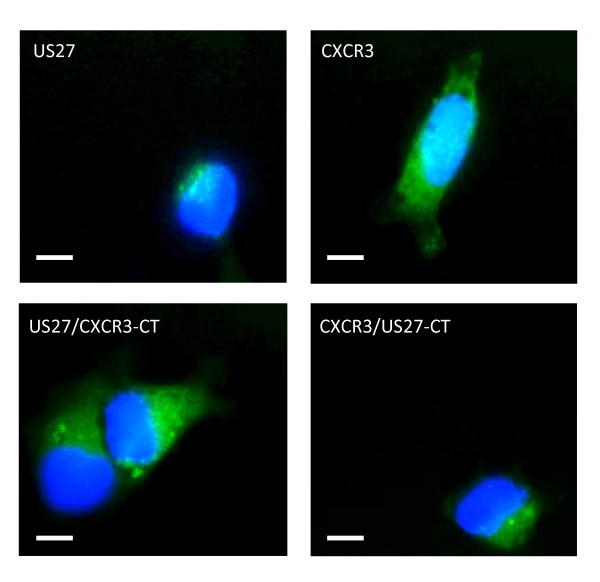
**US27 is found at the cell surface only transiently**. Stably transfected HEK293 cells were grown on glass coverslips in culture medium containing anti-FLAG antibodies for one hour, then washed, fixed, permeabilized, and stained with the appropriate secondary antibody. Scale bars = 10 μm.

In order to characterize the biological activities of the HCMV US27 receptor, we first constructed an expression plasmid encoding US27. The US27 gene from strain AD169 was cloned into the pcDNA3.1 vector, allowing for expression of the US27 protein as a fusion with a C-terminal *myc*/HIS epitope tag. US27 protein expression was investigated via immunofluorescence microscopy using human embryonic kidney cells (HEK293) grown on glass cover slips and transiently transfected with pcDNA-US27. After 48 hours, the cells were fixed, permeabilized, and stained with an anti-*myc *monoclonal antibody followed by a FITC-conjugated goat anti-mouse secondary antibody. The US27 protein (pUS27) was expressed in transfected cells and was found in a predominantly intracellular pattern (Figure 1A). Nuclei appeared blue due to DAPI staining, and the green fluorescence representing pUS27 was concentrated around the nucleus and in adjacent compartments. The epitope tag is unlikely to have altered distribution of pUS27, as transfection of HEK293 cells with either p3XFLAG-US27, which enabled expression of the protein with an N-terminal FLAG tag (Figures 2 and 3), or pEGFP-US27 (data not shown) resulted in the same intracellular localization pattern. These results are consistent with the perinuclear localization for pUS27 previously described in both transfected and infected cells by Fraile-Ramos et al. [42].

Given the striking intracellular localization of pUS27, we selected a more typical GPCR for comparison. CXCR3 is a human chemokine receptor and the expression pattern, tissue distribution, ligands, and signaling outcomes are well-characterized [44-47]. The CXCR3 gene was isolated from human peripheral blood mononuclear cells and cloned into pcDNA3.1. Immunofluorescence of transiently transfected HEK293 cells revealed that CXCR3 had a widespread distribution in which the complete outline of the adherent cell was visible, which is consistent with expression of this receptor on the cell surface (Figure 1B). When expressed with an N-terminal FLAG tag (Figure 3) or as an EGFP fusion protein (data not shown) CXCR3 also exhibited the same cell surface distribution.

To evaluate which domain of the US27 protein was responsible for the distinct intracellular localization pattern, a series of US27/CXCR3 receptor chimeras were created. The N-terminal extracellular domain of pUS27 (aa 1-34) was replaced with the corresponding extracellular domain of CXCR3, resulting in a fusion protein that contained amino acids 1-55 from CXCR3 and amino acids 34-362 of pUS27. Transient transfection of HEK293 cells with plasmid DNA encoding this chimera (pcDNA-CXCR3-NT/US27) revealed an intracellular distribution similar to that of wild-type pUS27 (Figure 1C). Likewise, a chimera consisting of aa 1-33 of US27 and 56-336 of CXCR3 (US27-NT/CXCR3) was also created, and this receptor retained the widespread surface distribution characteristic of CXCR3 (Figure 1D).

Next, a chimera consisting of amino acids 1-303 from pUS27 and the C-terminus of CXCR3 (aa 327-368) was prepared. This receptor chimera (US27/CXCR3-CT) was found to be expressed with a cell surface distribution similar to that of wild-type CXCR3 (Figure 1E). In contrast, a chimera containing amino acids 1-336 of CXCR3 and 304-362 of pUS27 (CXCR3/US27-CT) was found to exhibit intracellular localization similar to that of wild type pUS27 (Figure 1F). These findings strongly suggest that sequences within the C-terminal intracellular domain of HCMV US27 contribute to the intracellular receptor distribution.

**Figure 4:**
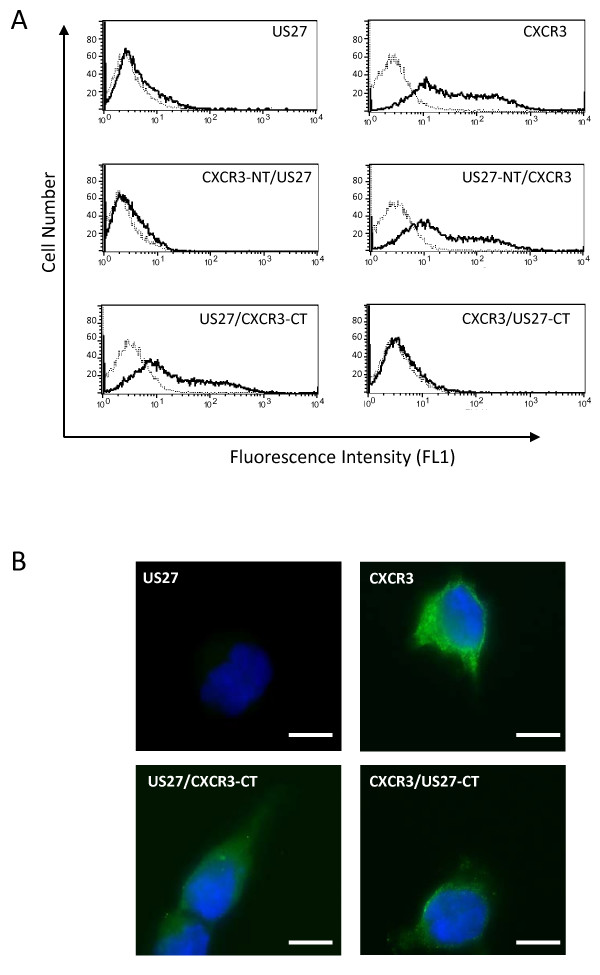
**Cell surface staining of US27**. A) HEK293 cells were transiently transfected with the indicated p3XFLAG constructs, harvested via gently scraping, staining with anti-FLAG antibodies, then examined via flow cytometry using a BD FACSCalibur. Data was analyzed using CellQuestPro. B) Transiently transfected cells grown on glass coverslips were fixed and stained in the absence of any detergents or permeabilization steps. After treatment with FITC-conjugated anti-mouse secondary antibodies, the coverslips were mounted with Prolong Gold containing DAPI. Scale bars = 10 μm.

Because of the significant differences observed in receptor localization, flow cytometry was performed to confirm whether receptors were found at the cell surface. HEK293 cells that were stably transfected with p3XFLAG constructs encoding US27, CXCR3, or chimeras were stained with anti-FLAG antibodies followed by FITC-conjugated secondary antibody. As shown in Figure 4A, cells stably transfected with p3XFLAG-US27 exhibited very little surface staining (solid lines) compared to cells stained with an isotype control antibody (dotted lines). Cells stably transfected with p3XFLAG-CXCR3 were found to express a significant amount of cell surface receptor. Chimeras containing swapped N-terminal domains (CXCR3-NT/US27 and US27-NT/CXCR3) exhibited staining that was markedly similar to the wild-type receptor (pUS27 and CXCR3, respectively). However, chimeras containing swapped C-terminal domains showed a significantly different expression pattern. The US27/CXCR3-CT receptor was detected on the cell surface, in direct contrast to the pattern observed for wild type pUS27, whereas the CXCR3/US27-CT receptor was not detected on the cell surface at all. These results confirm the receptor distributions observed when immunofluorescence staining was conducted in the absence of any permeabilization of the cells, as shown in Figure 4B. Almost no pUS27 was detected, confirming that this receptor is largely absent from the cell surface. Cells expressing CXCR3 could still be visualized even without permeabilization, demonstrating that this receptor is present on the cell surface. The chimeric receptors demonstrate the distribution predicted for the given C-terminal domain: US27/CXCR3-CT was clearly visible at the surface while CXCR3/US27-CT was virtually absent from the cell surface. These results indicate that the C-terminal domain of US27 conveys intracellular receptor localization.

To confirm that the C-terminal domain was necessary for intracellular localization of the viral receptor, a pUS27 deletion mutant lacking the C-terminal amino acids 304-362 (US27ΔCT) was expressed. This truncated receptor was found to display a more widespread cell surface distribution with a punctate and speckled appearance, similar to human CXCR3 (Figure 2). In order to pinpoint whether specific amino acids might mediate this intracellular localization, a series of successive truncations to the C-terminal domain of pUS27 were made. The US27Δ348, US27Δ338, and US27Δ322 mutants all displayed the same punctate but widespread surface distribution as the US27ΔCT protein (Figure 2). The same expression pattern was observed when the cells were stained in the presence or absence of permeabilization (data not shown). This could indicate that the amino acids mediating the intracellular localization of the US27 receptor are located at the very C-terminus of the protein between residues 348-362, since this region was absent from all of the deletion mutants examined. Interestingly, this region contains two leucine residues preceded by a series of acidic amino acids (amino acids 349-362 EEEFLL), which is a known sorting and internalization signal [48-50]. Thus, the results indicate that deletion of amino acids 348-362 ablates the intracellular localization seen for wild-type pUS27, possibly due to the loss of the di-leucine sorting motif.

**Figure 5:**
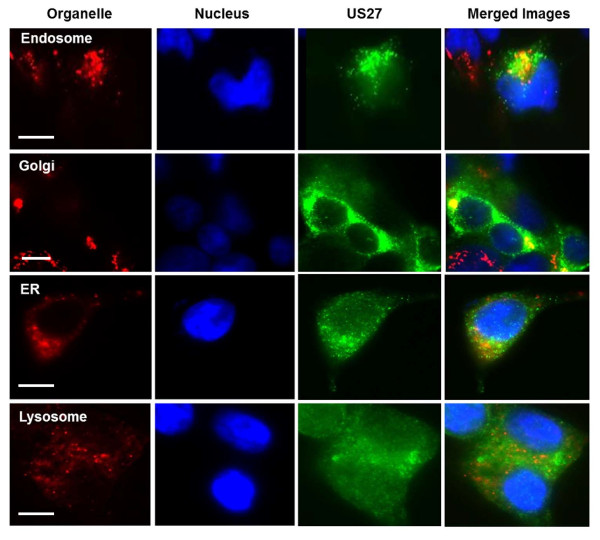
**US27 co-localizes with Golgi and endosome markers**. A stable p3XFLAG-US27 HEK293 cell line was stained with anti-FLAG antibodies and antibodies to the indicated organelles followed by FITC conjugated anti-mouse and TRITC-conjugated anti-rabbit secondary antibodies. Blue represents the DAPI-stained nuclei, green represents US27, and red represents organelles. The merged images result from imaging that compiles all three color images into one. Areas where green and red co-localize appear yellow. Scale bars = 10 μm.

To more precisely determine the subcellular location of wild type pUS27, HEK293 cells that stably expressed FLAG-tagged US27 proteins were analyzed by immunofluorescence microscopy. The US27 protein was found in compartments adjacent to the nucleus. These same compartments appeared red when labeled with an antibody specific for GMP130, a marker for cis-medial Golgi (Figure 5). A merged image revealed extensive co-localization of pUS27 with the Golgi apparatus, which is consistent with the observation that this protein is heavily glycosylated [38]. In addition, there was evidence that pUS27 was also present in the endosomes, which were stained with antibody to EEA1, early endosomal antigen 1. The distribution pattern was specific for the Golgi apparatus and endosomal compartments, as pUS27 did not significantly co-localize with a marker for the endoplasmic reticulum (calreticulin) or the lysosomes (LAMP2, lysosomal associated membrane protein 2). The results demonstrate that intracellular pUS27 is found mainly in the Golgi apparatus and endosomes.

**Figure 6:**
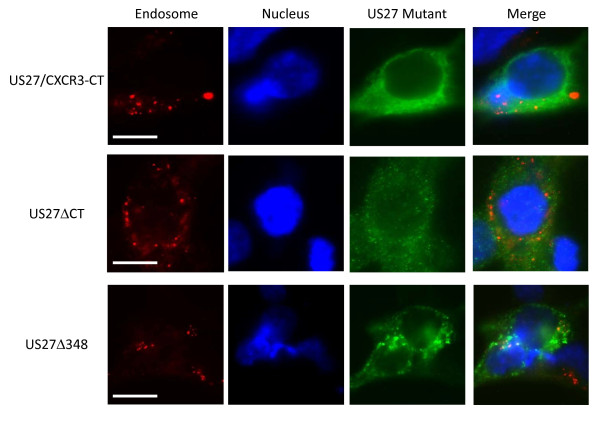
**US27 mutants do not co-localize with the endosome marker**. HEK293 cells were transfected with p3XFLAG constructs expressing the chimeric receptor US27/CXCR3-CT, deletion mutant US27ΔCT, or US27Δ348. Cells were co-stained with anti-FLAG and antibodies to the early endosomal antigen as described. Endosomes appear red, nuclei blue, and US27 mutants green. Scale bars = 10 μm.

In order to eliminate the possibility that pUS27 was simply retained inside the cell due to improper folding or other transport issues, antibody feeding experiments were performed. Briefly, stably transfected cells were cultured in the presence of anti-FLAG antibodies, then the cells were fixed, permeabilized, and stained with FITC conjugated secondary antibody. As shown in Figure 3, cells expressing pUS27 exhibit modest green fluorescence in the same perinuclear intracellular pattern observed previously (Figures 1, 2, and 5). This strongly indicates that the viral receptor is synthesized normally and transported to the cell surface, but it remains there only transiently before internalization. These findings support the notion that sequences in the C-terminal domain of pUS27 are responsible for the intracellular localization pattern. When the subcellular location of the US27/CXCR3-CT chimera was examined, however, there was no evidence of co-localization with endosomal markers (Figure 6). In addition, neither the US27ΔCT nor the US27Δ348 mutants were found to be concentrated in endosomal compartments. Altogether, these results demonstrate that the C-terminus of pUS27 is both necessary and sufficient for intracellular receptor localization to the endosomes.

## Discussion

HCMV has a remarkable capacity to institute life-long latent infections, largely as a result of having multiple mechanisms for modifying host immune responses. The virus expresses four proteins with homology to the GPCR superfamily, only one of which, US28, has been shown to function as a ligand-activated chemokine receptor to date.

In setting out to characterize the function of US27, we were first struck by the unusual cellular localization pattern. Most GPCRs are found in the plasma membrane, with an extracellular ligand binding domain displayed on the cell surface for access by ligands. In contrast, US27 was found predominantly inside the cell. Our results using transfected cells here are consistent with previous reports that have shown US27 to have an intracellular perinuclear distribution in both transfected and virus infected cells [42,51]. We further examined the distribution pattern by creating a series of receptor chimeras that contained domains of US27 and domains from the human chemokine receptor CXCR3. This receptor was originally selected as a fusion partner for use in a calcium signaling screen to identify possible chemokine ligands for US27. The closely related HCMV receptor US28 is relatively promiscuous and binds a large number of human chemokines, but US28 does not bind to any of the three known CXCR3 ligands (CXCL9/MIG, CXCL10/IP-10, and CXCL11/I-TAC). Thus, it was expected that using CXCR3 as a fusion partner was unlikely to result in identification of any false positive ligands for US27. In fact, the ligand screen failed to identify any human chemokine that caused calcium flux through wild type US27 or the US27/CXCR3-CT chimera (data not shown), but the unusual localization of the distribution of these receptors warranted further study.

Through immunofluorescence imaging of transiently transfected cells, wild-type pUS27 was found to be localized to intracellular compartments, whereas both the US27 fusion protein expressing the C-tail of CXCR3 (US27/CXCR3-CT) and US27 with a truncated C-terminus exhibited altered localization to the cellular surface (Figure 1E and 2, respectively). Moreover, the expression profile of US27/CXCR3-CT was more comparable to that of CXCR3 (Figure 1E, B). These findings are further supported by flow cytometry (Figure 4) and clearly demonstrate that the C-terminal amino acids of US27 are required for intracellular localization, since either removal or replacement of this domain ablates the intracellular distribution pattern.

Another chimera, CXCR3/US27-CT, was found mainly inside the cell, in a pattern that was distinct from that of wild-type CXCR3 but instead resembled that of wild-type pUS27. These results show that the C-terminal domain is sufficient to convert a cell surface receptor to an intracellular one. Likewise, a chimera that contained the N-terminal domain of CXCR3 and the remainder of the US27 receptor was found clustered around the nucleus inside the cell, further demonstrating that the C-terminal domain is what determines US27 receptor localization.

In a similar study, Waldhoer et al. [52] generated chimeric and mutant HCMV US28 proteins and found them to possess altered constitutive endocytic properties. The capacity of the C-terminal tail of US28 to mediate constitutive receptor endocytosis was transposable to membrane-localized GPCRs such as the endogenous NK_1 _receptor and ORF74 GPCR of HHV-8. Alternatively, replacing the C-tail of US28 with the C-terminal tails of the NK_1 _or ORF74 receptors yielded a predominant surface localization phenotype for the US28 receptor.

In the same study, removal of the C-tail of US28 was found to enhance signaling induced by fractalkine, perhaps due to increased availability of the receptor on the cell surface [52]. We have not yet had the opportunity to determine whether removal of the C-terminal domain of US27 impacts cell signaling, as there are no known ligands or signaling outcomes for US27 at this time. Interestingly, a previous report had suggested that US27 might bind to RANTES based on chemokine internalization studies with mutant viruses lacking either US27, US28, or both [14]. In this study, cells infected with wild type virus internalized RANTES whereas cells infected with a mutant lacking both US27 and US28 did not. Cells infected with virus mutants lacking either US27 or US28 internalized RANTES, albeit to a lesser extent than cells infected with wild type virus. However, RANTES binding to US27 has not yet been confirmed and we have observed no calcium flux or migration of HEK293 cells expressing either US27 or US27/CXCR3-CT in response to RANTES (data not shown).

We have demonstrated that the C-tail of HCMV US27 mediates intracellular localization, which is most likely due to interactions with cellular proteins. A previous study investigating the interactions between a library of C-terminal tails and various post-endocytic sorting proteins found that US27 associates with N-ethylmaleimide-sensitive factor, or NSF [53]. A hexameric ATPase, NSF is an adapter protein involved in post-endocytic sorting of GPCR. As shown here, the intracellular, perinuclear localization seen in the expression of wild-type US27 was replaced with more diffused and surface localized expression patterns due to truncations of the C-terminus (Figure 2). These findings suggest that the C-terminus of US27 is necessary for endocytic trafficking and the resultant predominant intracellular localization of the receptor, as likely mediated through the association of the C-tail with NSF or other post-endocytic sorting proteins.

Indeed, more detailed analysis of the C-terminal domain of US27 revealed that deletion of 14 amino acids from the C-terminus had the most pronounced effect on receptor localization (Figure 2). We are confident that this result is due to the loss of specific amino acids and not simply modification of protein folding or expression patterns, as we have produced numerous other chimeras, deletions, and point mutations that do not alter the distribution of pUS27 (data not shown). Furthermore, analysis of the amino acid sequence of the C-terminus of US27 using the Eukaryotic Linear Motif (ELM) resource [54] shows the presence of a known sorting and internalization signal, EEEFLL (amino acids 349-362). Di-leucine motifs mediate internalization from the cell surface and have been described for numerous proteins including the CD3 and CD4 proteins of T lymphocytes [48,49], interferon-γ receptor [55], opioid receptors [50], and the thrombin receptor PAR1 [56]. In addition, the Env and nef proteins of HIV have di-leucine motifs that are critical for virus replication and optimal infectivity [27,57]. Receptor internalization mediated by di-leucine motifs can be followed by either lysosomal targeting and receptor degradation or by accumulation in endocytic compartments and recycling back to the plasma membrane. The presence of an acidic amino acid residue four or five residues N-terminal to the di-leucine motif has been shown to result in inclusion in endocytic compartments and not targeting to the lysosomes [54]. The US27 sequence contains multiple acidic residues upstream of the di-leucine motif, which is consistent with our observations that the US27 receptor is infrequently found on the cell surface but instead localizes to the Golgi apparatus and endosomal compartments. A similar motif is found in the C-terminal domain of US28 (aa 301-306, ELHCLL) and has been shown to be responsible for mediating interaction of US28 with the AP-2 adapter complex [56]. Additional studies are underway to determine whether US27 binds directly to AP-2 and other intracellular sorting proteins. Interestingly, CXCR3 contains three leucine residues in the C-terminal domain (aa 332-334) but these are positioned near the cell membrane and do not appear to function as an endocytic sorting signal.

Due to the absence of an in vivo model for HCMV infection, the roles of US27 and US28 in the pathogenesis of HCMV infection in humans remains unclear. Given the numerous reports of US28 signaling activity, is seems that this protein must convey valuable immune evasion functions. For example, it has been suggested that US28 may act to scavenge inflammatory chemokines, removing them from the extracellular milieu and thus impairing leukocyte recruitment [14]. For US27, with no known ligands or signaling outcomes, the picture is even murkier. Viruses generally only retain genes that are beneficial to their survival, so it is likely that US27 serves an important, but as yet unknown, role in HCMV infection as well. Our results show that US27 is found predominantly inside the cell, and antibody feeding experiments confirm that the receptor does travel to the cell surface, only to be internalized again. Even in the absence of any chemokine binding, it is tempting to speculate that US27 could alter leukocyte trafficking of infected cells through endocytosis that results in the internalization of cellular chemokine receptors, rendering cells unresponsive to certain chemokine signals. In addition, recent studies have shown that US28 can form heterodimers with UL33 and UL78, and that heterodimerization can abrogate US28-mediated signaling [51]. Ongoing studies are aimed at investigating whether US27 forms dimers with other viral or cellular receptors and determining the true role of this receptor in virus infection and pathogenesis.

## Materials and methods

### Virus, cells, and antibodies

The AD169 strain of HCMV was used for these studies (ATCC, Manassas, VA). WI-38 human lung fibroblasts and human embryonic kidney cells (HEK293) were maintained in Eagle's minimum essential medium supplemented with 10% fetal bovine serum (Atlanta Biologicals, Atlanta, GA) in a humidified 37°C incubator with a 5% CO_2 _environment. Human peripheral blood mononuclear cells (PBMC) were isolated from the buffy coat of a healthy human donor and cultured in RPMI 1640 supplemented with 2 mM glutamine, 10 mM HEPES, 1 mM sodium pyruvate, 1.5 g/L sodium bicarbonate and 10% fetal bovine serum.

Anti-*myc *antibodies were obtained from Invitrogen (San Diego, CA) and the anti-FLAG antibody was from Sigma-Aldrich (St. Louis, MO). Organelle-specific antibodies were from GeneTex (Irvine, CA). The Golgi apparatus was detected with an antibody to Golgi Matrix Protein GM130, lysosomes were detected with an antibody to lysosomal-associated membrane protein 2 (LAMP2/CD107b), and endosomes were detected with an antibody to early endosome antigen 1 (EEA1). FITC-conjugated goat anti-mouse secondary antibodies, goat anti-rat TRITC and goat anti-rabbit TRITC secondary antibodies were from Anaspec (San Jose, CA). The endoplasmic reticulum was labeled via transfection using a Cell Painter plasmid (Origene, Rockville, MD) expressing RFP-calreticulin.

### Receptor constructs

The full length US27 gene was amplified via PCR using DNA harvested from AD169 infected cells at 14 days post-infection as a template, and the following gene specific primers: US27 forward 5'-CGGATCCATGACCACCTC TACAAATAATC-3' and US27 reverse 5'-GCTCGAGTTACAACAGAAATTCCTCCT C-3'. The resulting 1.1 kb PCR product was digested with BamHI and XhoI and then cloned into the corresponding sites of pcDNA3.1/*myc*-HisA (Invitrogen) to express the viral receptor with *myc *and 6XHis epitope tags at the carboxy-terminus. The full length US27 gene was also cloned into the HindIII and BamHI sites of p3XFLAG-CMV10 (Sigma-Aldrich) to generate the viral receptor with three adjacent FLAG epitopes at the N-terminus of the protein. Finally, full length US27 was cloned into the XhoI and BamHI sites of pEGFP-C1 (Clontech, Mountain View, CA) to create an EGFP-US27 fusion protein. DNA sequencing confirmed that the US27 sequence did not differ from the published sequence of the gene (Genbank accession #NC_001347, whole genome of HCMV strain AD169).

The full length gene for human chemokine receptor CXCR3 was amplified via RT-PCR from RNA isolated from human PBMCs using the following gene specific primers: CXCR3 forward 5'-CGGATCCATGGTCCTTGAGGTGAGTGAC-3' and CXCR3 reverse 5'-GCTCGAGTCACAAGCCCG AGTAGGAG-3'. The resulting 1.1 kb PCR product was digested with BamHI and XhoI and then cloned into the corresponding sites of pcDNA3.1/myc-HisA (Invitrogen) to express the cellular receptor with myc and 6XHis epitope tags at the carboxy-terminus. The full length CXCR3 gene was also cloned into the BamHI/HindIII sites of p3XFLAG-CMV10 and the HindIII/XhoI sites of pEGFP-C1 to create tagged fusion proteins as described above. DNA sequencing confirmed the CXCR3 sequence was the same as the published sequence (Genbank accession # AY242128).

Chimeric receptors consisting of segments of HCMV US27 fused to regions of human CXCR3 were created using a two-step PCR strategy. The 3' region of CXCR3 (nucleotides 979-1104) corresponding to amino acids 327-368 of the intracellular C-terminal domain was amplified using the CXCR3 reverse primer above with a forward primer containing an extended region that did not anneal to CXCR3 but was complementary to the 3' end of the US27 fragment (5'-GGCACTCAAATGAGGAAGGAGCGGATGTGGATGCTGCTC-3'). Likewise, the region of US27 between nucleotides 1-909 was amplified using the US27 forward primer and a reverse primer containing an extended region complementary to the 5' end of the CXCR3 fragment (5'-CAGCATCCACATCCGCTCCTTCCTCATTTGAGTGCCTAC-3'). These two fragments were the templates for a second round of PCR using the US27 forward primer and the CXCR3 reverse primer, resulting in a full length product containing nt 1-909 of US27 and nt 979-1104 of CXCR3. This 1 kb product was then purified, digested with restriction enzymes, and cloned into the BamHI/XhoI sites of the pcDNA3.1-*myc*/HisA vector. After transformation, DNA sequencing of a resulting positive clone confirmed it contained the sequence of US27 from nucleotides 1-909 and CXCR3 from 910 to 1032; this clone was designated US27/CXCR3-CT.

Three additional chimeras were produced using the same approach. CXCR3/US27-CT consisted of nucleotides 1-978 of CXCR3 and nucleotides 910-1089 of US27, CXCR3-NT/US27 comprised nucleotides 1-159 of CXCR3 and nucleotides 109-1089 of US27, and US27-NT/CXCR3 comprised nucleotides 1-108 of US27 and nucleotides 160-1032 of CXCR3. In addition, four US27 deletion mutants were created using PCR to truncate the coding sequence. US27ΔCT comprises nucleotides 1-908 of US27, US27Δ322 contains nucleotides 1-966, US27Δ338 contains nucleotides 1-1014, and US27Δ348 contains nucleotides 1-1044. Each chimeric gene or deletion mutant was cloned into the BamHI/XhoI sites of pcDNA3.1 and the HindIII/BamHI sites of p3XFLAG.

### Transient transfection and immunofluorescence microscopy

HEK293 cells were seeded in six-well dishes (2 × 10^5 ^cells per well) containing glass coverslips and then transfected with US27 or CXCR3 constructs and Fugene transfection reagent (Roche, Basel, Switzerland) according to the manufacturer's instructions. At 48 hours post-transfection, the cells were washed with PBS, fixed with 2% paraformaldehyde, permeabilized (where indicated) with 0.2 (w/v) Triton X-100, and then further treated with a mixture of 50% methanol-50% acetone. After blocking with PBS + 10% FBS, the cells were stained with primary antibody (1:250 dilution) at 37°C for one hour. After washing, the cells were incubated with fluorochrome-conjugated secondary antibody for one hour, washed again, then dried and mounted using Prolong Gold anti-fade reagent with DAPI (Invitrogen). Slides were viewed using a Zeiss A1 AxioObserver fluorescence microscope and image acquisition and analysis was performed using AxioVision software.

### Stable cell lines and co-localization experiments

HEK293 cells were transfected with p3XFLAG-US27 as described and then cultured in the presence of 1 mg/ml geneticin sulfate (Invitrogen) to select for stable transformants. After several weeks of continuous selection, a clonal cell line was prepared by limiting dilution assay and expression of FLAG-tagged US27 was confirmed via fluorescence microscopy following staining with anti-FLAG antibody. For co-localization studies, stable 3XFLAG-US27 cells were seeded onto glass coverslips and stained with anti-FLAG and the indicated organelle specific antibodies, followed by FITC-conjugated anti-mouse and TRITC-conjugated anti-rabbit secondary antibody. For co-localization with the ER marker, 3XFLAG-US27 stable cells were transfected with pCMV6-AC-RFP-calreticulin and stained with anti-FLAG and viewed 48 hours post-transfection. For antibody feeding assays, stably transfected HEK293 cells were grown on glass coverslips in medium containing the anti-FLAG antibody at 37°C for one hour, then fixed, permeabilized, and stained with FITC-conjugated anti-mouse secondary antibody prior to mounting with Prolong Gold anti-fade reagent with DAPI (Invitrogen).

### Flow Cytometry

HEK293 cells were transfected as described and 48 hours post-transfection cells were washed in PBS, then harvested and resuspended in FACS buffer (PBS + 1% BSA and 0.1% sodium azide) for surface staining with anti-FLAG antibody (1:100 dilution) followed by FITC-conjugated secondary antibody. Cells were stained for one hour on ice in the dark, then washed three times, fixed in 1% paraformaldehyde, and analyzed using a FACSCalibur and CellQuestPro software.

## Abbreviations

HCMV: human cytomegalovirus; GPCR: G-protein coupled receptor; RANTES: regulated on activation, normal T cells expressed and secreted; MIP-1α: macrophage inflammatory protein alpha; MAP kinase: mitogen activated protein kinase; NFAT: nuclear factor of activated T cells; CREB: cAMP response element-binding; NF-κB: nuclear factor kappa B; HHV-6: human herpesvirus 6; HHV-7: human herpesvirus 7; MCMV: murine cytomegalovirus; RCMV: rat cytomegalovirus; PBMC: peripheral blood mononuclear cells; EGFP: enhanced green fluorescence protein; PBS: phosphate buffered saline; BSA: bovine serum albumin; DAPI: 4':6-diamidino-2-phenylindole; FITC: fluorescein isothiocyanate; TRITC: tetramethylrhodamine isothiocyanate.

## Competing interests

The authors declare that they have no competing interests.

## Authors' contributions

LKS carried out the cloning of the receptor chimeras and some flow cytometry and immunofluorescence microscopy, KLA performed the antibody feeding experiments, and some flow cytometry and immunofluorescence microscopy, APL and TMD also carried out some immune fluorescence microscopy, and JVS conceived of the study, participated in its design and coordination and drafted the manuscript. All authors read and approved the final manuscript.

## Query

Q1: Figures: Journal requires that the first figure referenced in the manuscript text should be Figure 1. The second, Figure 2, etc. However, original sequence of the figure citations "Figure 1, Figure 3, and 5, Figure 2, Figure 4, Figure 5, and Figure 6" is out of order. Figure and citations were reordered so that they are cited in consecutive order. Please check if action taken is appropriate. Otherwise, kindly advise us on how to proceed.
